# Demand for integrative medicine among women in pregnancy and childbed: a German survey on patients’ needs

**DOI:** 10.1186/s12906-018-2249-y

**Published:** 2018-06-15

**Authors:** Nikolas Schürger, Evelyn Klein, Alexander Hapfelmeier, Marion Kiechle, Daniela Paepke

**Affiliations:** 1 Department of Gynecology and Obstetrics, Klinikum rechts der Isar, Technische Universität München, Munich, Germany; 2 Institute of Medical Informatics, Statistics and Epidemiology, Technische Universität München, Munich, Germany

**Keywords:** Complementary and alternative medicine (CAM), Psychological counseling, Nutritional counseling -- pregnancy, Childbed, Integrative medicine, Obstetrics

## Abstract

**Background:**

Although integrative medicine is gaining increasing attention and is claiming more and more its place in modern health care, it still plays a marginal role in conventional maternity care. The present study aims to examine the patterns of Complementary and Alternative Medicine (CAM) use and the demand for integrative therapies, including CAM, relaxation therapies, nutritional counseling, and psychological assistance, among women in pregnancy and childbed.

**Methods:**

The survey was conducted from April 2017 to July 2017 by means of a pseudo-anonymous 38-item questionnaire at the Department of Gynecology and Obstetrics, Klinikum rechts der Isar, Technical University of Munich. Eligible participants were women hospitalized due to pregnancy related complications and women in childbed. Descriptive statistics were generated to determine patterns of CAM use and demand for integrative therapeutic approaches. Univariate analysis was used to detect associations between patients’ characteristics and their interest in the different integrative therapies. Furthermore, binary logistic regression was used to estimate the odds ratio of demand for CAM.

**Results:**

A total of 394 out of 503 patients participated in the survey (78%). 60% declared using CAM in general, 45% specifically in relation to their pregnancy or childbed. Most commonly used modalities were vitamins (31% of all patients), yoga (24%), and herbal supplements (23%). Most popular sources of recommendation of CAM use were midwives and gynecologists. Integrative therapy options patients would have wanted alongside conventional maternity care were CAM (64%), relaxation therapies (44%), dietary counseling (28%), and psychological counseling (15%). Furthermore, associations between patients’ sociodemographic characteristics and their demand for integrative therapies were identified.

**Conclusions:**

The results of this study demonstrate that there is a considerable demand for integrative medicine and widespread use of CAM among women during pregnancy and childbed in Germany. Maternity health care providers should be aware of these findings in order to be able to better address patients’ needs and wishes. Our study findings should be interpreted with regard to patients in an hospital setting.

## Background

Integrative medicine (IM) seeks to promote a health care system that stands for the transition from the still dominant biomedical conception of health to a more biopsychosocial one. In fact, while conventional medical care often lays the focus on the measurable, physiological mechanisms that are responsible for disease development, an integrative approach takes account of the person as a whole, including mind, emotions, lifestyle, and body [1]. Although the role of these factors in maintaining both mental and physical well-being is extensively documented in the literature, they are often neglected in medical practice [2–5]. Therefore, IM aims at providing a more holistic vision on health care by incorporating ancillary and conventional therapeutic approaches and by laying great emphasis on addressing individual needs, patient empowerment, and patient autonomy [6]. Many of the interventions adopted by IM fall under the umbrella of Complementary and Alternative Medicine (CAM). The National Center for Complementary and Integrative Health (NCCIH) classifies CAM modalities into three categories: natural products (e. g. herbs, vitamins, minerals), mind and body-based practices (e. g. massage, yoga, meditation, acupuncture and osteopathic manipulation) and others (e. g. Ayurvedic medicine, traditional Chinese medicine, homeopathy) [7]. Nonetheless, the comprehensive spectrum of IM is broad and might encompass apart from CAM also nutritional counseling, psychological assistance, as well as various others. Although IM is increasingly accepted in oncology, which is substantiated by the growing number of integrative oncological programs [8], it undoubtedly plays a less important role in maternity care. However, it is noteworthy that especially CAM enjoys a high popularity among pregnant women [9, 10] and in their review of the international literature, Adams et al. noted that most studies with a sufficient sample size reported a prevalence of CAM use during pregnancy ranging between 20 and 60% [11]. Research suggests that childbearing women perceive conventional medicine as dangerous [12, 13], hence they often resort to CAM upon encountering pregnancy-related problems, believing it to be more natural and safe [14, 15]. In fact, a number of studies show that nonpharmacologic alternatives are widely used for nausea/vomiting, pain management, or labor induction [16–19]. On the one hand, certain CAM treatments have been proven to be effective and beneficial in pregnancy, including acupuncture for pelvic pain [20], omega-3 fatty acids for gestation and infant neurodevelopement [21], or perinatal yoga for depression and stress reduction [22, 23]. Muthukrishnan and colleagues demonstrated that maternal stress, strongly correlated with shorter gestation, preterm delivery and low birth weight [24, 25], could be significantly reduced by means of mindfulness meditation [26]. On the other hand, since the effect of many CAM modalities on maternal and fetal health are unknown and pregnant women frequently do not inform their pregnancy care provider about their complementary health practices [27, 28], uncritical CAM use has to be considered problematic. It is therefore of vital importance that physicians involved in maternity care should be aware of the usage patterns of childbearing women with regard to CAM. If applied with circumspection, however, CAM has the potential for reduction of drug consumption [29], medical expenditures [30] and significant cost savings [31]. On another note, there is plenty of research emphasizing the strong impact of the body mass index (BMI) on maternal and neonatal complications, such as gestational diabetes, pregnancy induced hypertension, macrosomia or preterm delivery [32–34]. Considering the rising incidence rates of maternal obesity [35], we strongly believe that nutritional counseling should have a more important role among maternity health care providers as well.

IM acknowledges the complexity of modern medical care and expands the spectrum of conventional health care. The primary purpose of our study was to assess the interest and demand for integrative therapeutic approaches among women during pregnancy and childbed. This information would enable health care providers to better address patients’ needs and wishes during that time. Moreover, we zoomed in on CAM and examined its prevalence of use during pregnancy and childbed, its most commonly applied methods, and the most popular sources of recommendation, in order to gain additional information on patterns of CAM usage in this particular population group.

## Methods

Over a period of 3 months from April to July 2017 the survey was conducted by means of a structured questionnaire at the Department of Gynecology and Obstetrics, Technical University of Munich (TUM), Klinikum recht der Isar, in Munich, Germany.

### Study population

The questionnaires were handed out to hospitalized pregnant women (pregnancy group) and to women after their delivery (childbed/postpartum group) at our Department of Gynecology and Obstetrics. Inclusion criteria for participation were age 18 years or older, command of the German or English language, and the mental ability to fill out a questionnaire. The questionnaires were handed out to all the patients who met these criteria, disregarding stage of gestation, risk profile or hospitalisation time. Altogether, 162 questionnaires were distributed to the prepartum group and 341 to the postpartum group. Since our two study population groups represent two different stages on the common trajectory of pregnancy, we decided to include them both in the survey and to highlight possible differences concerning the demand for IM.

### Questionnaire

The survey questionnaire, written in the German language, comprised a total of 38 items and was developed by the authors based on previous research in the field of gynecological oncology. To avoid comprehension problems and to test the survey process, we pretested the set of closed-ended questions using a convenience sample of 10 pregnant and childbed patients. The revised, final questionnaire consisted of three sections:

- Assessment of personal use, opinions, and sources of information regarding CAM
- Attitudes, interest and demand for integrative therapeutic approaches during pregnancy and childbed, namely:
- CAM (defined according to the NCCIH) [7].
- Relaxation therapies: yoga, meditation, autogenic training, qigong/tai chi.
- Dietary counseling.
- Psychological counseling.

Due to their growing popularity, we took the liberty of looking at the health approaches which primarily address the promotion of mental and psychological well-being (b) separately. Traditionally included in the CAM family, these constitute a separate integrative therapy subgroup in this paper, referred to as “relaxation therapies”. By doing so, we provided a basis for comparison between relaxation therapies and the rest of the CAM group, as well as between relaxation therapies and the other key player that deals with psychological needs, psychological counseling.

3. Assessment of sociodemographic factors and health behaviors including age, education, marital status, employment, BMI and physical activity

Patients from the pregnancy group answered the questions with regards to their pregnancy. Patients from the postpartum group with regards to their pregnancy and childbed, depending on the question.

Participants were informed that participation was voluntary and that all data were collected strictly in pseudonymous form. Those who chose to participate gave their informed consent by filling out the questionnaire. The questionnaires were filled out autonomously. However, if they were only English-speaking or preferred being assisted, the survey was conducted in a face-to-face interview.

### Ethical approval

The final version was approved by the Ethics Committee of the Technical University of Munich (TUM) with the project number 73/17 S.

A preliminary statement on the front of the questionnaire informed the patients about the voluntary and pseudonymous nature of the study as well as its purpose. Respondents were not offered any incentive for study participation.

### Statistical analysis

Descriptive statistics such as absolute and relative frequencies as well as means and standard deviations were generated to determine the prevalence and patterns of CAM use, as well as the demand for integrative therapeutic approaches. Univariate t-tests and chi-squared tests were conducted for hypothesis testing on associations between interest in integrative therapy options and the application of CAM, on the one hand, and patients’ sociodemographic characteristics and health behaviors, on the other. Moreover, using logistic regression, we estimated the odds ratio of the demand for CAM, adjusted for patients’ characteristics. Our approach towards data analysis was exploratory, without a specific a priori hypothesis to prove. Patients with missing values were excluded from the analysis of the corresponding variables. Hypothesis testing was conducted on two-sided 5% significance levels. Data management and statistical analyses were performed using the statistical software SPSS, Version 20 (IBM Corp., Armonk, N.Y., USA).

## Results

In total, 133 out of 162 pregnant patients (82%) and 261 out of 341 (76%) postpartum patients participated in the study (overall return rate of 78%). The mean age of the study population was 32.0 years (standard deviation [SD] 5.0 years). The sociodemographic characteristics of the participants are summarized in Table 1.

**Table 1:** General characteristics of the study population (*n* = 394)

Characteristics	*n*	%
Age		
18–27	68	17
28–37	274	70
38–48	52	13
BMI		
Normal weight (18–25)	275	70
Underweight (< 18,5)	14	4
Overweight (25–30)	68	17
Obese (> 30)	37	9
Marital status		
Unmarried	66	17
Married/partner	328	83
Employment		
Unemployed	89	23
Employed	305	77
Education		
No graduation/secondary modern school (9 years)	77	20
Secondary modern school (10 years)	61	15
Grammar school	256	65
Physical activity		
Never/sometimes	233	59
1-2× per week	115	29
3-4× per week/daily	46	12
CAM use		
No CAM user	158	40
CAM user	236	60

**Fig. 1:**
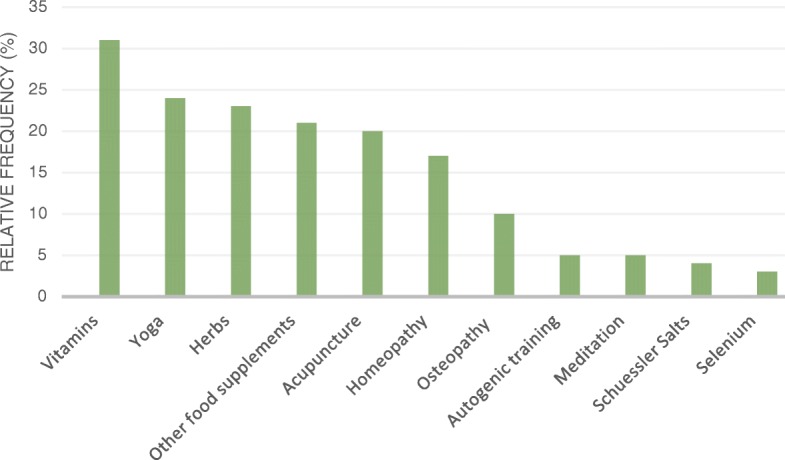
Relative frequencies of most commonly used CAM modalities and relaxation therapies during pregnancy. Relative frequencies are reported in relation to the total sample size (*n* = 394). Multiple responses were possible

Overall, 60% (*n* = 236) reported using CAM in general, and 45% (*n* = 177) specifically in relation to their pregnancy or childbed. The frequencies of most commonly used CAM modalities and relaxation therapies are depicted in Fig. 1. Vitamins were the category patients used most (31%, *n* = 122), followed by yoga (24%, *n* = 94) and herbs (23%, *n* = 89).

**Fig. 2:**
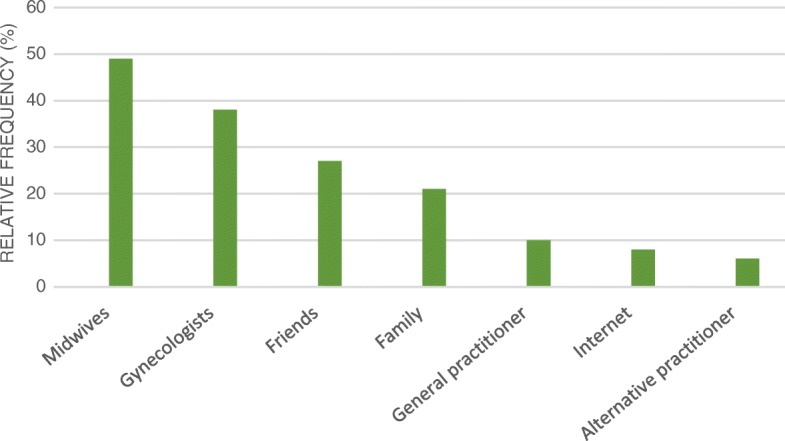
Sources of recommendation which had the strongest influence on participants using CAM specifically in relation to their pregnancy or childbed (*n* = 177). Multiple responses were possible

When asked who recommended the CAM therapies, almost half (49%) of the participants who were using it specifically in relation to their pregnancy or childbed reported being influenced by their midwives, followed by their gynecologist (38%), friends (27%) and family (21%). The least popular sources of information and recommendation appeared to be the Internet (8%) and alternative practitioners (6%) (Fig. 2).

Table 2 shows survey participants’ interest in and wish for integrative therapeutic approaches during pregnancy and childbed alongside conventional maternity care. Altogether, more than half of the respondents (64%, *n* = 251) wanted CAM implemented into maternity care and would have taken advantage of it, had it been part of the regular treatment services. Analogously, 44% declared themselves in favor of relaxation therapies (*n* = 174), 28% of dietary counseling (*n* = 112), and 15% of psychological counseling (*n* = 60). Furthermore, we compared the two groups, women in childbed and currently pregnant women, with each other regarding their wish for integrative therapies. Overall, every fifth pregnant woman (*n* = 27) would have welcomed psychological assistance during their stay in our Department of Gynecology and Obstetrics, compared to every eighth woman in childbed (*n* = 33). Univariate testing did not reveal any statistically significant level of difference between the two groups regarding the other integrative health approaches.

**Table 2:** Comparison between women in childbed and pregnant women regarding their demand for integrative therapy options

Demand for integrative therapeutic approaches	All respondents (*n* = 394)	Women in childbed (*n* = 261)	Pregnant women (*n* = 133)	*p*-value
Demand for CAM (%)				0.615
	63.7	62.8	65.4	
	(*n* = 251)	(*n* = 164)	(*n* = 87)	
Demand for relaxation therapies (%)				0.423
	44.2	45.6	41.4	
	(*n* = 174)	(*n* = 119)	(*n* = 55)	
Demand for dietary counseling (%)				0.964
	28.4	28.4	28.6	
	(*n* = 112)	(*n* = 74)	(*n* = 38)	
Demand for psychological counseling (%)				0.045
	15.2	12.6	20.3	
	(*n* = 60)	(*n* = 33)	(*n* = 27)	

In the following, we analyzed which factors might be related to the use of CAM and the demand for integrative therapies. Univariate analysis was used to detect associations between application of CAM methods and demand for the three most popular integrative therapeutic approaches -CAM, relaxation therapies, dietary counseling-, on the one hand, and patients’ sociodemographic characteristics and health behaviors, on the other (Table 3).

**Table 3:** Univariate analyses of patients’ characteristics associated with CAM use and demand for integrative therapeutic approaches

	CAM use			Demand for CAM			Demand for relaxation therapies			Demand for dietary counseling		
Characteristics	Users (*n* = 236)	Non-users (*n* = 158)	*p*-value	Interest (*n* = 200)	No interest (*n* = 194)	*p*-value	Interest (*n* = 174)	No interest (*n* = 220)	*p*-value	Interest (*n* = 112)	No interest (*n* = 282)	*p*-value
Age ± SD	32.8 ± 4.5	30.9 ± 5.4	*p* < 0.001	32.7 ± 4.6	31.0 ± 5.5	*p* = 0.036	32.3 ± 4.8	31.9 ± 5.2	*p* = 0.412	31.9 ± 4.8	32.1 ± 5.1	*p* = 0.683
BMI ± SD	23.7 ± 4.2	24.4 ± 4.9	*p* = 0.102	23.6 ± 4.4	24.7 ± 4.6	*p* = 0.042	24.3 ± 5.3	23.8 ± 3.8	*p* = 0.267	24.3 ± 4.6	23.9 ± 4.5	*p* = 0.354
Marital status (%)			*P* = 0.013			*p* = 0.070			*p* = 0.085			*p* = 0.174
Married/partner	87.3	77.8		86.1	79.0		79.9	86.4		89.5	85.1	
Unmarried	12.7	22.2		13.9	21.0		20.1	13.6		20.5	14.9	
Employment (%)			*p* < 0.001			*p* < 0.001			*p* < 0.001			*p* = 0.378
Employed	89.4	59.5		86.9	60.8		86.8	70.0		80.4	76.2	
Unemployed	10.6	40.5		13.1	39.2		13.2	30.0		19.6	23.8	
Education (%)			*p* < 0.001			*p* < 0.001			*p* = 0.001			*p* = 0.136
No graduation/secondary modern school (9 years)	7.6	37.3		8.4	39.2		13.2	24.5		19.6	19.5	
Secondary modern school (10 years)	15.3	15.8		14.3	17.4		12.1	18.2		9.8	17.7	
Grammar school	77.1	46.8		77.3	43.4		74.7	57.3		70.5	62.8	
Physical activity (%)			*p* < 0.001			*p* < 0.001			*p* = 0.311			*p* = 0.272
Never/sometimes	51.3	70.9		52.9	69.9		56.3	61.4		58.9	59.2	
1-2× per week	35.6	20.3		37.1	16.1		33.3	26.4		25.9	30.9	
3-4× per week/daily	13.1	8.9		10.0	14.0		10.3	12.3		15.2	9.9	
CAM use (%)						*p* < 0.001			*p* = 0.015			*p* = 0.835
CAM user				72.1	38.5		66.7	54.5		60.7	59.6	
Non-CAM user				27.9	61.5		33.3	45.5		39.3	40.4	

On average, CAM use was more frequent among participants who were older, employed, married or living with a partner, who had higher levels of education, and who were engaging regularly in physical activity.

Increased interest in and demand for CAM during maternity care was associated with patients who, in general, were older, employed, CAM users, weighing less, and who had higher levels of education. Altogether, the women demanding relaxation therapies showed to be more often employed, CAM users and to have more frequently higher levels of education.

Furthermore, participants with higher levels of education were more often interested in psychological counseling than participants with lower degrees of education (Table 3).

Table 4 presents the results of our binary logistic regression model as odds ratios of the demand for CAM in maternity care. Compared to Non-CAM users, women using CAM had 2.59 times higher odds (95% CI, 1.58–4.27; *p* < 0.001) of endorsing CAM as a regular service program during maternity care. Moreover, education was confirmed to be significantly associated with the demand for CAM. Participants who had attended grammar school had the highest odds of advocating the implementation of CAM into conventional maternity care compared to participants with less schooling. Women without any schooling or only the mandatory 9 years had the lowest odds of demanding CAM.

**Table 4:** Bivariate logistic regression model with odds ratio of demand for CAM, adjusted for participants’ characteristics

	Demand for CAM			
Characteristics	Odds ratio	95% CI		*p*-value
		Lower	Upper	
Age				0.650
18–27	1.00 (reference)			
28–37	1.34	0.71	2.52	
38–48	1.36	0.57	3.23	
BMI				0.152
Normal weight (18–25)	1.00 (reference)			
Underweight (< 18, 5)	4.15	0.76	22.57	
Overweight (25–30)	1.61	0.84	3.09	
Obese (> 30)	0.78	0.35	1.75	
Marital status				1.000
Unmarried	1.00 (reference)			
Married/partner	1.00	0.53	1.89	
Employment				0.313
Unemployed	1.00 (reference)			
Employed	1.40	0.73	2.71	
Education				
No graduation/secondary modern school (9 years)	1.00 (reference)			0.001
Secondary modern school (10 years)	2.53	1.13	5.66	
Grammar school	4.20	2.00	8.84	
Physical activity				0.078
Never/sometimes	1.00 (reference)			
1-2× per week	1.77	0.98	3.20	
3-4× per week/daily	0.58	0.29	1.18	
CAM use				< 0.001
No CAM user	1.00 (reference)			
CAM user	2.59	1.58	4.27	

## Discussion

In this study, we surveyed nearly 400 German women in pregnancy and childbed investigating their patterns of CAM use and their demand for integrative therapeutic approaches. The main findings are that the majority of our participants were using CAM, with dietary supplements being the most prevalent category. Most influential recommenders of CAM appeared to be midwives and gynecologists. Furthermore, a large number of respondents would welcome the integration of integrative health approaches into conventional maternity care.

The prevalence of CAM use during pregnancy varies widely throughout the published literature. Adams et al. critically reviewed the international literature on the subject published between 1999 and 2008 and found that most of the studies with sufficient sample size reported a prevalence rate between 20 and 60% [11]. Pallivalappila et al. on the other hand systematically reviewed the primary literature from 2008 to 2013 and found the prevalence to range between 6 and 74% [36]. The discrepancies between the single studies partly stem from the lack of a standard, consistent definition of CAM and associated modalities, as well as differences in culture, ethnicities and study design. Hence, despite the fact that our prevalence estimates fall into the range reported by previous papers, they are not directly comparable.

Pallivalappila et al. further pointed out that the definition of CAM provided by the WHO is rather vague and open to interpretation. Since many research papers, instead, adopted the definition proposed by the National Center for Complementary and Integrative Health (NCCIH) [37–41], we followed suit, deeming it concise and comprehensive. In order to establish a uniform and standardized definition of CAM, we suggest that future researchers follow our example.

The frequencies of the most commonly used CAM methods during pregnancy and postpartum period are subject to broad variation in the international literature as well within our study cohort. However, most surveys on that matter report dietary supplements, including vitamins, herbs, etc., as the most prevalent category of CAM [11, 39, 42, 43]. In view of the literature, our reported frequencies of vitamin and herbal supplement use (31 and 23% respectively) are comparatively low [11, 44]. This might be due to the fact that the use of vitamins and especially herbal remedies is less deeply rooted in German culture and society compared to other countries. While there is evidence for the efficacy and safety of some CAM modalities during pregnancy or delivery [20–23, 26, 45], the wide use of botanicals can pose some risks. Considering the potential adverse effects associated with the use of some herbal remedies and due to the lack of information concerning the safety of many others [46–48], further clinical trials are needed to investigate the innocuousness and adequacy of the most commonly used herbal supplements. Ideally, maternity care providers should be able to provide evidence-based information on the various CAM methods in the future, recommending only those which have been proven to be effective and without hazard to maternal or foetal health.

Our findings that midwives and gynecologists were the most popular sources of information and recommendation regarding CAM use (49 and 38% respectively) are partially consistent with the literature [40, 42, 49]. Nonetheless, other researchers reported that pregnant women appear to be more influenced by non-professional recommendations such as from personal experience or friends and family than by professional ones [11, 28, 43, 48, 50]. Studies suggest that, to some extent, this might be due to women’s perceived or experienced negative attitude towards CAM among professional healthcare providers [51, 52]. The relatively poor use of the Internet or alternative practitioners as a source of information has been confirmed by previous research [28, 43, 50].

To the best of our knowledge, so far, no studies have been undertaken to assess the demand for integrative therapeutic approaches among women in childbed and pregnancy.

More than half of our survey participants (64%) responded that they would welcome the integration of CAM into conventional maternity care. However, it is of note that CAM users had 2.59 times higher odds of demanding it, implying that those who were in favor of CAM and already using it on a general basis, are very likely to be the same who want it to be implemented. Moreover, our regression model reveals that higher levels of education increased the likelihood of demand for CAM, as has been reported by other researchers in regard to the use of CAM [37–39].

More than a quarter of our study population (28%) expressed their wish for dietary counseling. Although the association between high maternal BMI and complications both for mother and child are well known, the incidence of gestational diabetes mellitus (GDM) is increasing worldwide [32–34, 53]. A Finnish research group studied the effects of nutritional counseling on the eating habits of Finnish pregnant women at higher risk for GDM and found it to be very effective [54]. Offering dietary counseling during pregnancy to women with risk factors could fight the rise of GDM and reduce its detrimental effect on maternal and fetal health.

Pregnancy and childbirth are temporary episodes in a woman’s life, often associated with heightened levels of joy but also worries and anxiety. A woman who is hospitalized due to pregnancy-related problems will undoubtedly be experiencing immense distress, especially if the life of the child is threatened. This fact probably accounts for the increased demand for psychological assistance among the pregnant group compared to the childbed group. However, in cases where the newborn must be moved to a neonatal intensive care unit or the woman develops a postpartum depression (PPD), childbed can quickly become a period of enormous psychological strain, too. Prevalence estimates for PPD vary between 9.6 and 13% [55–57], whereas the postpartum blues, defined as a mild and transient mood disturbance occurring shortly after delivery, has prevalence rates of up to even 80% [58]. While conventional, biomedical medicine predominantly focuses on the treatment of physical and tangible problems, our data show a clear demand for therapeutic interventions aiming at the promotion of mental and psychological well-being. Nearly half of the women surveyed (44%) stated that they would have taken advantage of relaxation therapies, if they had been offered to them during their stay in our Department of Gynecology and Obstetrics, and expressed the wish for their integration. Analogously, 15% of the participants stated interest in psychological counseling. Despite the number of studies throwing light on the relationship between the antenatal psychological health and clinical outcomes [24, 25, 59, 60] and despite the demonstrated efficacy of some interventions such as yoga, mindfulness mediation or psychological assistance [22, 23, 26, 61], the psychological dimension of care still plays a minor role in conventional maternity care.

In line with previous research, we can confirm that age, education, marital status, employment and physical activity play an essential role regarding the decision on CAM use [11, 37–39, 42, 43]. High education, employment and usage of CAM also increase the likelihood of demanding for CAM and relaxation therapies, suggesting that patients’ sociodemographic characteristics typically associated with CAM use, may also be found in those who are generally in favor of integrative supportive services.

Our findings demonstrate a widespread use of CAM as well as a keen interest in integrative therapeutic approaches among our patients in pregnancy and childbed. Maternity care providers should be aware that, due to their unique situation, often associated with helplessness and worries, their patients might have particular needs which go beyond the spectrum of conventional medical care. IM offers a more patient-centered care which addresses their necessities more adequately. Hence, we believe that maternity health care professionals should receive basic training regarding integrative health approaches in order to be able to discern and pay due attention to their patients’ individual needs.

Our study results should be considered in light of some limitations. Firstly, survey data was collected mainly based on self-report and may therefore be subject to recall bias. Secondly, we conducted the survey in one hospital only in Munich, Germany, and consequently the results may not hold true for other parts of the country. Thirdly, especially the pregnant group of our sample might introduce some bias concerning their opinion on the integration of integrative therapeutic approaches. As mentioned before, practically all of them were inpatients due to pregnancy-related complications and hence their stress levels were rather high. Understandably, their demand for the single integrative therapies evaluated might surpass that of pregnant women who did not encounter any problems during their gravidity. Thus, their wish for integration should be interpreted with regard to the conventional maternity care provided by a hospital rather than by resident gynecologists or other resident physicians.

## Conclusions

The incorporation of integrative health approaches into the predominantly biomedical health system will play an important role in the medical care of the future. While to some extent IM has already established itself in medical fields such as oncology, it still plays a marginal role in conventional maternity care. Our data emphasises the demand for integrative medicine among women during pregnancy as well as childbed. Maternity health care professionals should be aware of this in order to be able to better address patients’ needs. Ideally, attending physicians should receive training and dispose of sound expertise regarding integrative therapies.

## Abbreviations

BMI: Body mass index
CAM: Complementary and Alternative Medicine
GDM: Gestational diabetes mellitus
IM: Integrative Medicine
NCCIH: The National Center for Complementary and Integrative Health
PPD: Postpartum depression
SD: Standard deviation
TUM: Technical University of Munich
WHO: World Health Organization
